# Model‐informed exploration of the boundaries of safe aluminium exposure from allergen immunotherapy in children

**DOI:** 10.1111/pai.70181

**Published:** 2025-08-26

**Authors:** Karin Weisser, Gaby Wangorsch, Niklas Hartung, Wilhelm Huisinga, Brigitte Keller‐Stanislawski

**Affiliations:** ^1^ Division Safety of Biomedicines and Diagnostics Paul‐Ehrlich‐Institut (Federal Institute for Vaccines and Biomedicines) Langen Germany; ^2^ Institute of Mathematics University of Potsdam Potsdam Germany; ^3^ Paul‐Ehrlich‐Institut (Federal Institute for Vaccines and Biomedicines) Langen Germany

**Keywords:** adjuvants, allergen immunotherapy, aluminium, computer simulation, immunologic, patient safety, toxicokinetics

## Abstract

**Background:**

The safety of aluminium (Al) exposure from medicinal products for subcutaneous allergen immunotherapy (SCIT) is still under debate due to their administration in many doses over years. Especially for children, model‐informed risk assessment is urgently needed in the absence of clinical study data.

**Methods:**

We applied a physiologically‐based toxicokinetic Al model for simulation of Al exposure from various SCIT scenarios in children (5 and 10 years) compared to adults (35 years) in addition to continuous Al exposure from dietary intake.

**Results:**

Simulations of a worst‐case Al exposure scenario (five‐year SCIT; four‐weekly injections; 1.25 mg Al per dose) in children reveal substantial, but subtoxic and transient increases in bone Al content. Predicted increases of Al levels in the brain appear negligible. In both of these storage organs, Al levels predicted at 50 years of age were only marginally affected by the previous SCIT treatment. Two exceptional SCIT scenarios, prolongation of treatment duration to 40 years and parallel treatment with two 1.25 mg Al‐containing products, were associated with elevated bone Al levels above the normal adult range and close to a level where potential harm to bone metabolism cannot be excluded.

**Conclusions:**

Simulations support the tolerability of single SCIT treatments in all age groups ≥5 years, also with respect to the long‐term Al body burden. The Pharmacopoeial limit of 1.25 mg Al per dose could be seen as a conservative threshold also for the sum of doses from parallel treatments. The decision to prolong therapy with Al‐containing SCIT products should be taken under careful consideration of Al accumulation.

**Key message**

These are the first quantitative physiologically‐based model‐informed predictions of the Al increase in relevant tissues after treatment with Al‐adjuvanted SCIT products in children compared to adults. Simulations suggest that single SCIT treatments are tolerable in all groups ≥5 years of age. However, therapy decisions for prolongation or parallel treatments should be taken under careful consideration of Al accumulation. The results provide for the first time a scientific basis for regulatory risk assessment and therapy decisions regarding the use of Al‐adjuvanted SCIT products in children. They are considered to be of substantial interest for clinicians and scientists in the field of pediatric allergy.

## 1 INTRODUCTION

For many years, aluminium (Al) hydroxide (AH) has been used as an adjuvant in allergen products for subcutaneous (s.c.) immunotherapy (SCIT). SCIT is a long‐established treatment method for inhalant and insect venom allergies in the pediatric population.^1^ Most of the products authorized in the EU are recommended from the age of 5 years onward.

European guidance documents on allergen immunotherapy^2^, ^3^, ^4^, ^5^ emphasize the relevance of the pediatric population and recommend early treatment. According to epidemiological data from Germany a substantial portion of children suffering from allergic rhinitis are treated with SCIT, for example, 30% in the age group of children between 11 and 17 years.^6^ With respect to insect venom allergy around 10% of children and adolescents with systemic sting reactions (SSR) on hymenoptera stings are treated with SCIT.^7^ This suggests a considerable number of children and adolescents under SCIT exposure.

As laid down by the European Pharmacopoiea,^8^ the highest possible Al dose in SCIT products is 1.25 mg per single human dose. Multiple (four‐weekly) s.c. dose applications during a single perennial full‐dose maintenance therapy can add up to about 15 mg of Al absorbed per year. The cumulative Al dose can be even higher when two or more SCIT therapies are combined. A substantial contribution of SCIT to the overall Al exposure in humans was recently proven in adult patients by an increase in urinary Al excretion during SCIT compared to controls.^9^

Al is prone to accumulate in the body when protective gastrointestinal mechanisms are bypassed by parenteral administration, when renal function is impaired (patients with renal compromise and neonates), or when exposure is high (long‐term total parenteral nutrition (TPN)). Signs of systemic Al toxicity notably occur in bone, liver, and the central nervous system^10^, ^11^ There is consensus that Al is a contributor to osteomalacia and encephalopathy, which were reported in the 1970s and 1980s as secondary to high aluminium exposure from Al contaminated fluids in dialysis patients or patients receiving TPN.^11^, ^12^, ^13^ Al has also been shown to increase the risk of parenteral nutrition‐associated liver disease (PNALD).^14^

Even though there is no signal from pharmacovigilance or clinical studies, concerns on the safety of Al in SCIT products are still raised. Hence, there is an urgent need to explore the boundaries of safe Al exposure. It has long been stated that a physiologically‐based pharmacokinetic model would be crucial to predict the risk from various aluminium exposures.^15^ A physiologically‐based approach would be essential especially for predictions in children in order to reflect pediatric growth and maturation. These considerations and the lack of toxicokinetic data of Al from adjuvants gave reason for starting a concerted aluminium toxicokinetics project in 2015 (reviewed recently^16^). During this project we demonstrated for the first time in rats that Al absorbed from SCIT applications reaches bone^17^ which indicated that this source of exposure may raise concerns of systemic toxicity. In addition, we successfully developed a physiologically‐based toxicokinetic (PBTK) model for Al built on a comprehensive animal and human 26Al toxicokinetic data set.^18^ An extended PBTK model has recently been published after further substantial development and validation steps were taken, with particular efforts to make it applicable for children and Al‐adjuvanted medicinal products.^19^ The model accounts for changes in physiology and maturation from birth to adulthood. It also includes a novel physiological bone submodel for the kinetics of Al as a bone‐seeking element by leveraging data on age‐dependent bone calcium (Ca) metabolism.^20^ Subcutaneous absorption rates from insoluble AH used as adjuvant have been implemented, both for products adjuvanted by addition of commercial AH (e.g., Alhydrogel) or by generation of AH “in situ” via precipitation in the presence of allergens.

Here we leverage for the first time the extended PBTK model to predict and assess Al exposure from adjuvanted allergen products with respect to specific age groups and various therapeutic scenarios.

## 2 METHODS

### 2.1 Simulation scenarios

#### 2.1.1 Long‐term Al exposure from dietary intake (“FOOD ONLY”)

A continuous age‐adjusted dietary intake as the main source of Al in humans was simulated to generate background exposure levels of Al expected in otherwise healthy SCIT patients. Based on results of European diet studies,^21^ a mean value of 0.8 mg Al/kg/week was chosen for children and adults (>12 months) resulting from consumption of food and water, and 0.1, 0.2, 0.4, and 0.8 mg/kg/week for infants aged 0–3, 4–6, 7–9, and 10–12 months, respectively, resulting from infant formulae and other foods manufactured especially for infants. An average oral bioavailability of 0.17%, as well as initial Al levels at birth, built up in all organs during embryonal development, were assumed as explained in Hartung et al.^19^ We used this dietary intake scenario (“FOOD ONLY”) as the basis for simulations of additional Al exposure from various SCIT scenarios (“FOOD+SCIT”).

#### 2.1.2 Additional Al exposure from SCIT (“FOOD+SCIT”)

Al exposure from SCIT was simulated as a “worst‐case “perennial full‐dose maintenance treatment” in three age groups: children (age at start of SCIT: 5y, ‘child’”), adolescents (10 years, “adol”), and adults (35y, “adult”). Due to negligible Al amounts in preceding updosing schedules (using high dilutions of SCIT doses), these were omitted from the simulation. The highest possible Al amount per human dose (1.25 mg, “high”) according to the European Pharmacopoeia^8^ and the shortest dosing interval recommended in product informations in Europe (4 weeks, 28 days) were assumed. Treatment duration and adjuvant type (commercial Alhydrogel‐like (“Ahy”) or in situ prepared AH (“Ains”)) were varied as described below.

##### Single five‐year SCIT treatments

Simulations for all three age groups were conducted assuming a treatment duration of 5 years (being the longest duration recommended in most authorized SCIT products in the EU), and using the *Ahy*‐adjuvant type (referred to as *Child‐high‐Ahy‐5, Adol‐high‐Ahy‐5, Adult‐high‐Ahy‐5, respectively*). Each scenario corresponded to a total number of 65 full doses with a cumulative amount of 81.25 mg Al. The simulation in children was repeated using in situ prepared AH (*Child‐high‐Ains‐5*) to explore the influence of the adjuvant type.

##### Prolonged SCIT treatments

Consecutive administration of two high‐dose, five‐year SCIT treatments was simulated in children (*Child‐high‐Ahy‐2* × *5*) corresponding to 130 full doses with a cumulative amount of 162.5 mg Al. As treatment with insect venom allergy products in special cases (high risk patients) can be extended up to even lifelong duration, we also simulated a SCIT over 40 years starting at 10 years of age (*Adol‐high‐Ahy‐40*; 520 full doses and 650 mg Al).

##### Parallel SCIT treatment

Parallel administration of two high‐dose, five‐year SCIT treatments was simulated in the child age group (*Child‐2xhigh‐Ahy‐5*; 2 × 65 doses and 162.5 mg Al).

### 2.2 PBTK model structure and simulation details

All simulations were based on the PBTK model reported in Hartung et al.^19^ This model is characterized by a consistent age‐dependent physiological parametrization, combined with a specific model for GFR (glomerular filtration rate) describing maturation after birth, and a new dynamic physiological bone submodel. In this submodel, Al uptake into bone is assumed to be proportional to calcium (Ca) uptake, and release from bone is identical to that of Ca. The Ca kinetic functions, as obtained in Hartung et al.^20^ captured age‐dependent bone remodeling processes during childhood and specifically the peak during puberty.

Inter‐individual variability is implemented on model parameters (oral bioavailability, GFR and tissue distribution).^19^ The model further contains dosing modules for oral and parenteral routes of administration. For s.c. absorption of Al from adjuvants, a specific zero‐order absorption rate was used for each adjuvant type (*Ahy*: 0.0024864/day, *Ains*: 0.0082392/day) estimated from injection site data in animal studies (see^19^). We assumed the absorption rates to be lognormally distributed in the population, with a 50% coefficient of variation, as was estimated for other compounds administered s.c.^22^

Simulation results and statistical analyses were obtained using the software R, version 4.2.2^23^ specifically using the R package mlxR version 4.2.0^24^ for Al exposure predictions in tissues and plasma. We used an identical set of *N* = 500 female individuals in each simulation to ensure comparability among all scenarios. We considered female subjects as the toxicologically more sensitive population after preliminary simulations for both sexes had revealed slightly higher tissue Al levels in females (see Figure S1).

### 2.3 Data retrieval and evaluation

All predicted Al concentration‐time curves are displayed as median with lower (5% percentile) and upper (95% percentile, p95) bounds. Al concentrations (median, p95) in plasma and tissues were calculated both 5 years after the start of each SCIT and at the age of 50. A 90% confidence interval (90% CI) was calculated for p95 as an estimate for the toxicologically relevant upper bound of the population. By capturing the impact of sampling on the predicted p95, this 90% CI demonstrated the adequacy of the chosen number of 500 simulated individuals. We graphically superimposed the total exposure levels resulting from FOOD+SCIT on the median exposure expected from FOOD ONLY (dotted line) to highlight the increase in Al exposure attributed to SCIT.

Three different ratios of p95 levels reached in bone or brain after FOOD+SCIT compared to FOOD ONLY were calculated (see Table 1): *p95 ratio 5/5* as a measure for the increase 5 years after the start of SCIT, *p95 ratio 5/50* for comparison of peak levels 5 years after the start of SCIT to normal adult levels at age 50 after sole food exposure, and *p95 ratio 50/50* as a measure for the long‐term impact of a SCIT on the Al body burden accumulated at age 50.

**TABLE 1:** Aluminium concentrations predicted in plasma, bone, and brain in a female population after average dietary Al intake only (“FOOD ONLY”), or in combination with a SCIT treatment (“FOOD + SCIT”; see Section 2).

Age group (age at start of SCIT)	Al exposure scenario	Al concentration 5y after start of SCIT											Al concentration at age 50					
		plasma (μg/L)			bone (μg/g ww)				brain (μg/g ww)				Bone (μg/g ww)			Brain (μg/g ww)		
		m	p95 (90%‐CI)	p95 ratio 5/5	m	p95 (90%‐CI)	p95 ratio 5/5	p95 ratio 5/50	m	p95 (90%‐CI)	p95 ratio 5/5	p95 ratio 5/50	m	p95 (90%‐CI)	p95 ratio 50/50	m	p95 (90%‐CI)	p95 ratio 50/50
Adult (35 years)	FOOD ONLY	1.4	6.1 (5.5–8.2)	–	0.6	5.2 (4.7–6.1)	–	–	0.23	0.75 (0.70–0.93)	–	–	0.6	5.4 (4.9–6.3)	–	0.28	0.94 (0.87–1.19)	–
	FOOD + SCIT *Adult‐high‐Ahy‐5*	2.2	7.1 (6.6–9.2)	1.2	0.8	5.5 (5.0–6.4)	1.0	1.0	0.24	0.76 (0.71–0.95)	1.0	0.8	0.7	5.6 (5.1–6.6)	1.0	0.29	0.95 (0.88–1.22)	1.01
Adole‐scent (10 years)	FOOD ONLY	1.2	4.9 (4.6–6.4)	–	0.5	3.5 (2.9–4.0)	–	–	0.11	0.28 (0.26–0.33)	–	–	0.6	5.4 (4.9–6.3)	–	0.28	0.94 (0.87–1.19)	–
	FOOD + SCIT *Adol‐high‐Ahy‐5*	2.0	6.2 (5.7–7.1)	1.3	0.9	4.5 (4.3–4.9)	1.3	0.8	0.12	0.29 (0.28–0.34)	1.0	0.3	0.7	5.4 (5.1–6.5)	1.0	0.29	0.96 (0.89–1.23)	1.02
	FOOD + SCIT *Adol‐high‐Ahy‐40*	2.0	6.2 (5.7–7.1)	1.3	0.9	4.5 (4.3–4.9)	1.3	0.8	0.12	0.29 (0.28–0.34)	1.0	0.3	1.1	7.0 (6.4–8.1)	1.3	0.38	1.11 (1.03–1.37)	1.18
Child (5 years)	FOOD ONLY	0.9	3.8 (3.6–4.9)	–	0.4	3.0 (2.5–3.4)	–	–	0.09	0.23 (0.21–0.25)	–	–	0.6	5.4 (4.9–6.3)	–	0.28	0.94 (0.87–1.19)	–
	FOOD + SCIT *Child‐high‐Ahy‐5*	2.0	5.4 (5.0–6.2)	1.4	1.0	4.8 (4.3–5.2)	1.6	0.9	0.12	0.25 (0.24–0.28)	1.1	0.3	0.6	5.4 (5.0–6.4)	1.0	0.30	0.97 (0.90–1.25)	1.03
	FOOD + SCIT *Child‐high‐Ains‐5*	1.9	5.3 (5.0–6.4)	1.4	1.0	4.8 (4.5–5.3)	1.6	0.9	0.12	0.25 (0.24–0.28)	1.1	0.3	0.6	5.4 (5.0–6.3)	1.0	0.30	0.97 (0.91–1.25)	1.03
	FOOD + SCIT *Child‐high‐Ahy‐2* × *5*	2.0	5.4 (5.0–6.2)	1.4	1.0	4.8 (4.3–5.2)	1.6	0.9	0.12	0.25 (0.24–0.28)	1.1	0.3	0.7	5.5 (5.1–6.5)	1.0	0.32	0.98 (0.94–1.28)	1.04
	FOOD + SCIT *Child‐2xhigh‐Ahy‐5*	2.9	7.0 (6.6–7.8)	1.8	1.5	7.0 (6.2–8.0)	2.4	1.3	0.14	0.28 (0.26–0.31)	1.2	0.3	0.6	5.5 (5.0–6.4)	1.0	0.33	1.00 (0.96–1.30)	1.06

Abbreviations: m, Median; p95, 95%‐percentile; p95 ratio 5/5, P95(FOOD+SCIT) 5 years after start of SCIT/p95 (FOOD ONLY) at the same timepoint; p95 ratio 5/50, P95(FOOD+SCIT) 5 years after start of SCIT/p95 (FOOD ONLY) at age 50; p95 ratio 50/50, P95(FOOD+SCIT) at age 50/p95 (FOOD ONLY) at age 50.

The toxicological evaluation of predicted total Al levels in tissues was based on data of upper limits of normal (ULN) or critical levels of Al in tissues reported in the literature. If values were published as μg/g dry weight (dw), we converted them into Al amounts per wet weight (ww) to allow for a meaningful comparison with predicted Al tissue levels modeled as ww. For example, the conversion factor for bone samples (0.497) accounts for the difference between Al in dry (and usually bone marrow‐free) bone samples and predicted Al concentrations in wet model bone tissue defined as cartilage‐free and bone marrow‐including (for details see,^19^ chapter 2.2.3).

A level of 5 μg/g ww was considered as ULN for Al content in the bone of healthy adults (converted from the ULN of 10 μg/g dw reviewed by^13^). Levels >30 μg/g ww are reported to be clearly associated with Al‐induced bone disease and osteomalacia.^13^, ^25^, ^26^ The lowest Al content in bone associated with osteomalacia symptoms (7 μg/g ww, herein referred to as “critical level”) was found in one patient on long‐term TPN (converted from 14 μg/g dw,^27^).

A derivation of an ULN is more uncertain for brain tissue. A median of 0.2 μg Al/g ww is reported from post mortem brains of healthy adults (overall age range 55–105 years^28^, ^29^) with a clear trend of an increase with age.^30^ A range of 0.02–1 μg/g ww for ‘normal’ brains over several decades is reported (converted from 0.1 to 4.5 μg/g dw^31^; conversion factor 0.23, see,^19^ chapter 2.2.3). Therefore, we herein refer to 1 μg/g ww as an ULN for Al in brain, given the available data. A critical level of Al content in brain with respect to neurotoxicity is unknown.

A normal Al concentration in healthy adults at steady state of 1 (0.5) μg/g ww is reported for liver (kidney) tissue.^15^ A ULN of ca. 4 (1.6) μg/g ww for adults was deduced from the range of 0.07–4.33 (0.04–1.57) μg/g ww measured in 140 adult autopsy liver (kidney) tissues in Germany (age range 21–81 years^30^). Measurements in children showing hepatotoxic symptoms after TPN were in the range of 8–40.5 μg Al/g ww (converted from 32 to 162 μg/g dw^32^); however, the observed hepatic pathology was not clearly related to TPN only. Therefore, critical levels for the Al content in liver (or kidney) also cannot be inferred.

All ULN levels derived here are displayed in the respective figures as a horizontal dotted line.

## 3 RESULTS

### 3.1 Long‐term Al exposure in humans from dietary intake (“FOOD ONLY”)

**FIGURE 1:**
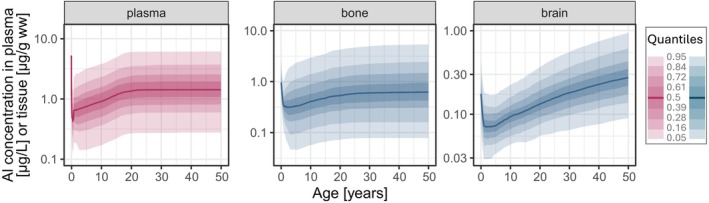
Predictions of Al concentration time courses in plasma, bone, and brain from birth to adulthood following an average dietary Al exposure (“FOOD ONLY”) in a female population (solid line: Median; shaded areas: Quantiles).

**FIGURE 2:**
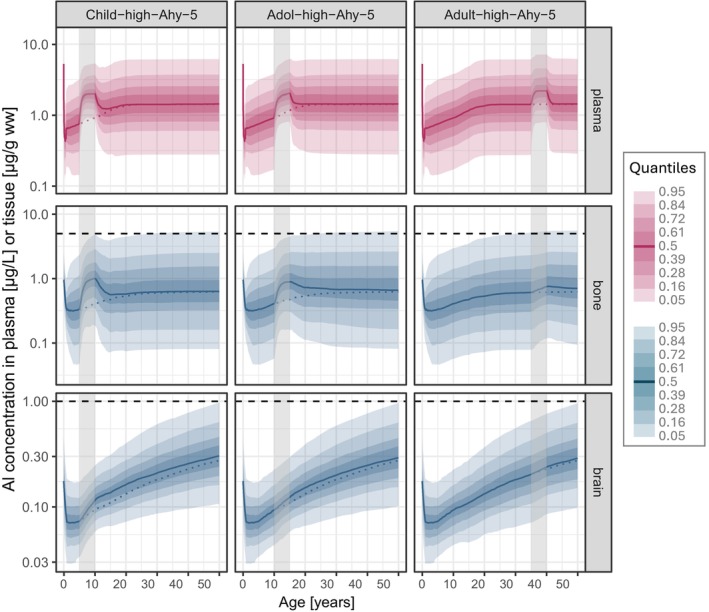
Predictions of Al concentration time courses in the liver following all simulated SCIT scenarios (see Figures 3 and 4) in addition to the continuous background dietary exposure (solid line: Median; shaded areas: Quantiles; vertical shaded box: Time period of SCIT treatment; horizontal dashed line: Upper limit of normal (see Section 2); dotted line: Median time course of “FOOD ONLY” exposure (upper left panel)).

Time courses of Al concentrations predicted in plasma, bone, and brain following continuous average dietary Al intake in females from 0 to 50 years of age are displayed in Figure 1. High initial Al levels in neonates at birth immediately decreased due to the dilution effect of body growth and the low Al intake via baby food. After this period, Al levels steadily increased. In adulthood, due to the end of body growth and a constant Al intake, a steady state was reached in plasma and in all well‐perfused and rapidly exchanging organs (e.g., liver, Figure 2). Steady state in bone was not reached until late adulthood due to a slower release rate. Levels in brain accumulated continuously since brain acts as a sink.

Al levels predicted in plasma, bone, and brain at relevant timepoints for comparison within each SCIT age group are shown in Table 1 (“FOOD ONLY”). Since p95 of adults predicted in bone (5.2 μg/g ww) matches well with the ULN of 5 μg/g ww reported in literature for adults, the corresponding p95 values after FOOD ONLY exposure in children at 10 and 15 years of age (3.0 and 3.5 μg/g ww, respectively) could serve as estimates of “pediatric ULN” for Al in bone.

### 3.2 Additional Al exposure from SCIT (“FOOD+SCIT”)

#### 3.2.1 Single SCIT treatments

**FIGURE 3:**
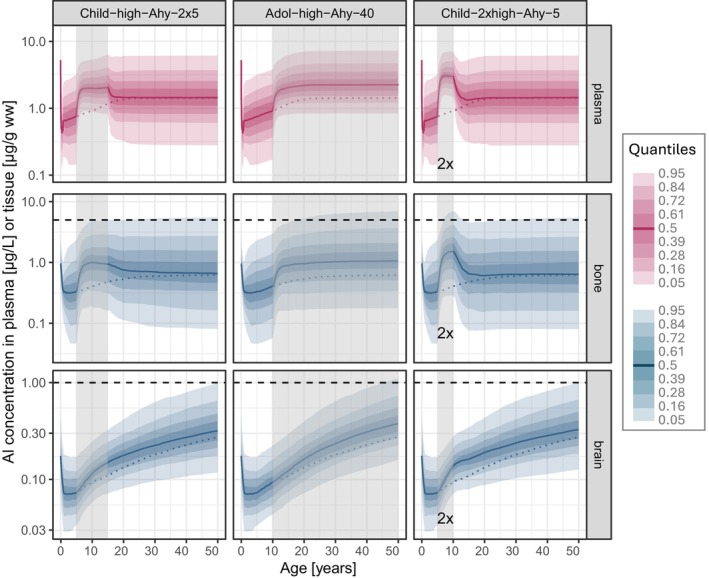
Predictions of Al concentration time courses in plasma, bone, and brain following a 5‐year SCIT treatment (Alhydrogel adjuvant type) during childhood (*Child‐high‐Ahy5*; left panel), adolescence (*Adol‐high‐Ahy5*; middle panel) and adulthood (*Adult‐high‐Ahy‐5*; right panel), each in addition to the continuous background dietary exposure (solid line: Median; colored shaded areas: Quantiles; vertical shaded box: Time period of SCIT treatment; horizontal dashed line: Upper limit of normal (ULN; see Section 2); dotted line: Median time course of “FOOD ONLY” exposure (see Figure 1)).

Figure 3 shows the Al concentration time courses predicted after a five‐year SCIT treatment in children (*Child‐high‐Ahy5*), adolescents (*Adol‐high‐Ahy5*), or adults (*Adult‐high‐Ahy5*), each in addition to the Al food intake. Total Al levels reached 5 years after the start of each SCIT and at the age of 50 are summarized in Table 1.

The five‐year high dose SCIT treatment using the commercial Alhydrogel‐like adjuvant led to an increase in Al plasma steady state levels during the SCIT period in all age groups, with the highest rise from 3.8 to 5.4 μg/L (p95; ratio 1.4) in children. A parallel increase was predicted in bone Al content, forming a pronounced peak during SCIT. Maximum Al levels (p95) in bone at the end of SCIT amounted to 1.0 μg/g ww in children, 0.9 in adolescents, and 0.8 in adults, which was, respectively, 1.6, 1.4, and 1.0 times the baseline level at that timepoint (p95 ratio 5/5). All peak levels during SCIT were still at or below the level predicted in a female adult after 50 years of FOOD ONLY (p95 ratio 5/50: 0.9, 0.8, 1.0 μg/g ww, respectively).

The increase in bone Al content induced by SCIT was transient. There was a slow but almost complete return to baseline levels in plasma and bone after about 5 years (children) to 10 years (adolescents and adults) after the end of SCIT. Al levels in bone reached at age 50 in all age groups did not differ from those after FOOD ONLY (p95 ratio 50/50: 1.0).

Only a small increase in brain Al content during SCIT compared to background exposure was predicted in all age groups (max p95 ratio 5/5: 1.1). The quantities added were still present at 50 years of age, reflected by a p95 ratio 50/50 of 1.01–1.03.

Al levels also increased in liver (Figure 2) and kidney (see Figure S2). The highest Al levels in liver and kidney during a single SCIT were reached in children with a median (p95) of 0.10 (1.6)μg/g ww each.

Al levels predicted after a five‐year SCIT in children with a product containing an in situ prepared AH adjuvant (*Child‐high‐Ains‐5*) were very similar to the levels reached with the commercial AH product (Table 1). Only the rise of the Al peak in plasma and bone after the start of SCIT was steeper (see Figure S3, vs. Figure 3, left panel), in line with the higher absorption rate for the in situ prepared adjuvant (see Section 2).

#### 3.2.2 Prolonged SCIT treatments

**FIGURE 4:**
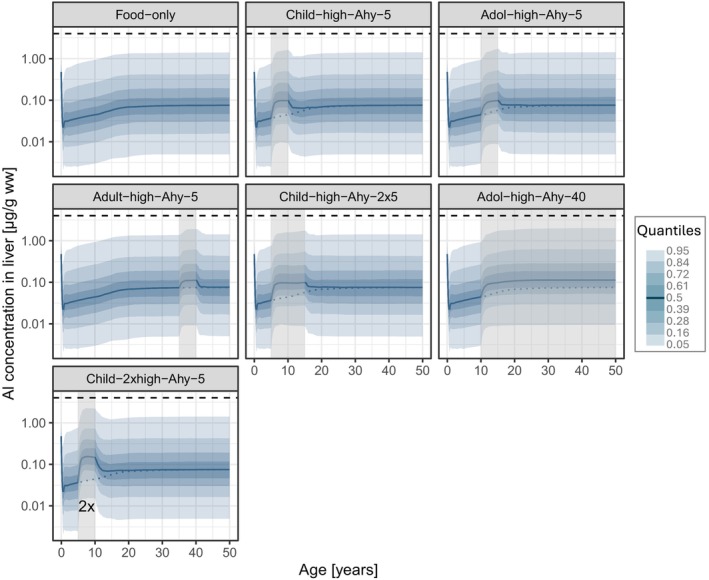
Predictions of Al concentration time courses in plasma, bone, and brain following SCIT treatments (Alhydrogel adjuvant type), either two consecutive 5‐year treatments during childhood (*child‐high‐Ahy‐2* × *5*; left panel), or “lifelong” treatment starting in adolescence (*Adol‐high‐Ahy‐40*; middle panel), or (C) two parallel 5‐year treatments during childhood (*child‐2xhigh‐Ahy5*; right panel), each in addition to the continuous background dietary exposure (solid line: Median; colored shaded areas: Quantiles; vertical shaded box: Time period of SCIT treatment; horizontal dashed line: Upper limit of normal (see Section 2); dotted line: Median time course of “FOOD ONLY” exposure (see Figure 1)).

Two consecutive five‐year SCIT treatments in children (Figure 4, left panel) yielded maximum Al levels during SCIT similar to the single five‐year treatment (Figure 3 and Table 1). Due to the prolongation, elevated levels were maintained for a longer period, and the return to baseline in plasma and bone was postponed by an additional 10 years from 20 (single SCIT, Figure 3) to 30 years of age.

Effects on long‐term Al levels were still small. Accumulated brain Al level at the age of 50 increased by 4% compared to FOOD ONLY exposure (p95: 0.98 μg/g ww, p95 ratio 50/50: 1.04; Table 1).

Further prolongation of treatment (40 years) starting at 10 years of age (Figure 4, middle panel) led to a similar initial increase of Al in bone as with the shorter treatment (Figure 3), but levels continued to accumulate throughout the treatment. Median Al bone content at age 50 was increased twofold; from age 20 onwards p95 exceeded ULN, reaching the critical level of 7.0 μg/g ww at age 50. In brain, p95 at age 50 slightly exceeded “ULN” due to an increase of 18% (p95: 1.1 μg/g ww, p95 ratio 50/50: 1.18; Table 1).

#### 3.2.3 Parallel SCIT treatment

The parallel treatment with two SCIT products (Figure 4, right panel) showed markedly higher peak Al levels than after the single SCIT treatment in the same individuals: Median plasma levels at the end of the five‐year treatment reached 2.9 μg/L (p95: 7.0 μg/L), p95 bone Al levels rose to 7.0 μg/g ww (p95 ratio 5/5: 2.4; Table 1) exceeding p95 at age 50 after FOOD ONLY exposure (p95 ratio 5/50: 1.3; Table 1). As it did after single treatment (Figure 3) return to baseline occurred at ca. 20 years of age and, consequently, the long‐term effect on levels in bone at age 50 also remained negligible (p95 ratio 50/50: 1.0). In contrast, brain Al levels were slightly elevated throughout childhood. Brain Al levels were 6% higher at the age of 50 than without treatment (p95 ratio 50/50: 1.06) compared to a 3% increase after a single treatment. The highest Al levels in liver and kidney reached a median (p95) of 0.15 (2.2) μg/g ww each (Figure 2 and Figure S2, respectively).

## 4 DISCUSSION

We have presented the first model‐based predictions of the additional Al exposure resulting from subcutaneous allergen immunotherapy (SCIT) using Al‐adjuvanted products. As there are no toxicokinetic data available from clinical studies, model‐informed assessment is of major importance, especially since therapeutic schemes are highly variable and are subject to change without having a measure to evaluate possible changes in risk.

It is important to emphasize that the predictions are based on a wide range of clinical and physiological data that were used to build and validate the model. All assumptions regarding initial Al levels at birth, physiological growth, Al absorption, and Al bone kinetics following the human age‐dependent Ca turnover have been thoroughly validated.^19^ In particular, proof has been shown^19^ of the model's qualification for making credible predictions for lifelong peroral Al exposure from food as well as for s.c. exposure from Al adjuvanted SCIT products after either single dose application in rats^17^ or multiple dose administrations during SCIT in human adults.^9^

Predictions of worst‐case single five‐year SCIT scenarios showed only moderate elevations of the steady state Al level in plasma in all age groups. This is attributed to the slow s.c. absorption rates of poorly soluble AH. The highest predicted median level (3 μg/L) remained within average normal levels of adults (1–3 μg/L^15^). Nevertheless, Al levels in bone increased in parallel. The increase was most pronounced in children, with an almost twofold increase relative to background level. At the end of SCIT, 14.4% of the children are predicted to exceed p95 of the background exposure, that is, the “pediatric 10y‐ULN” (3.0 μg/g ww), compared to 5% without therapy. P95 predicted in children at the end of SCIT is close but still within normal levels in adults accumulated after 50 years of dietary intake. Due to the bone remodeling process (particularly extensive during childhood and adolescence) this increase is transient, even though return to background levels takes years. In children, it can take as long as the SCIT period itself. Hence, levels in bone at the age of 50 will only be marginally affected by a SCIT treatment in childhood. Al levels in brain were also only marginally affected, both during SCIT and at the age of 50, although brain is modeled as a sink compartment. This is due to a very slow brain uptake rate, which had been estimated based on available data.^19^ The plausibility of the brain kinetics in our model is supported by the finding that the predicted median level of 0.28 μg/g ww accumulated after 50 years of realistic food intake matches well with reference levels in literature.^28^, ^29^ In conclusion, the impact of a single five‐year SCIT in childhood or adolescence on the Al burden in bone and brain in late adulthood appears to be negligible.

This also holds true for treatments with products using in situ prepared AH as an adjuvant. With regard to peak Al levels reached in tissues, the overall impact of faster s.c. absorption (duration ca. 4 months) from each injection site compared to commercial AH like Alhydrogel (duration ca. one year^17^) was shown to be marginal.

Two exceptional SCIT scenarios were associated with elevated bone Al levels above 5 μg/g ww (ULN) and close to a level considered critical: parallel treatment reaching peak levels during the SCIT period in childhood and single lifelong treatment reaching its maximum later in adulthood due to continuous accumulation. Parallel treatment was the only scenario where the peak bone Al level during SCIT exceeded that in healthy adults after 50 years of FOOD ONLY exposure (p95 ratio 5/50: 1.3). In both scenarios, 5% of the individuals were predicted to exceed a bone Al content of 7 μg/g ww (p95 = 7.0) which relates to the lowest reported level associated with osteomalacia in TPN patients^27^ (see Section 2). The safety margin to unambiguously toxic levels (30 μg/g ww) is about fourfold. It should be noted that the role of Al as a causative factor for bone disease in TPN patients is not well defined and other factors may contribute.^27^, ^33^ Evidence is limited and primarily based on small studies published in the 1980s and 1990s.^11^

In all SCIT scenarios, the additional increase in brain Al levels was predicted to be very small, due to a low uptake rate of Al into the brain compared to other organs. Predicted total levels did not exceed the presumed ULN (1 μg/g ww; see Section 2), and even the highest exposure scenarios (parallel and lifelong treatment) only reached brain Al content increases of 6% and 18%, respectively. However, it is acknowledged that estimates of ULN in the brain are uncertain and based on limited data.

During high Al exposure, the liver is also an organ of potential Al toxicity. The p95 value of 2.2 μg/g ww predicted for the highest dose scenario (parallel treatment) is within normal levels of adults (ULN: 4 μg/g ww) and considerably (fourfold) lower than the lowest level measured in children showing hepatotoxic symptoms after TPN (8 μg/g ww^32^; see Section 2). Similar to bone, it has to be taken into account that in TPN patients, Al is presumably not the sole cause for hepatotoxicity.^32^ Since the kidney is not a major target organ and Al‐induced nephrotoxicity has not yet been described in humans,^10^ the predicted parallel increase of Al levels in the kidney is not classifiable.

We simulated Al exposure from SCIT in addition to a continuous Al background exposure. The presumed background intake was based on approximate overall estimates for Europe and could regionally be lower or even higher. For children and adults, it corresponded to 80% of the TWI (tolerable weekly intake; 1 mg Al/kg/week^21^). There are indications that overall Al exposure in Europe is close to or even exceeds TWI.^34^ As the resulting Al time courses (including predicted variability) in plasma and tissues matched well with the “normal” ranges of Al levels in literature,^19^ including very recent German urine data in the control group of Hiller et al.,^9^ we considered our intake scenario suitable to reflect a realistic range of background Al exposure (from whatever source).

Apart from their allergy, we focused on otherwise healthy subjects. Simulations were performed until age 50, since our model does not account for renal impairment or other alterations impacting Al kinetics that could appear at advanced age. The considered scenarios reflect worst‐case examples of perennial four‐weekly treatments with the highest possible Al dose of 1.25 mg. Products authorized in the EU contain from 0.19 to 1.13 mg Al per maintenance dose. The product‐specific aluminium content can be obtained from the summary of product characteristics (Section 2 – Qualitative and Quantitative Composition). This implies that for many products, dose‐adjusted simulations would lead to lower Al increases. While our model is dose‐linear, predicted time courses would, however, not scale relative to the therapeutic dose, since the simulations are in addition to the baseline food intake that would not change. The model assumed 100% bioavailability of Al from the injection site, which also is a conservative approach. On the other hand, due to the almost exclusive renal elimination of Al, renal impairment in children or adults (beyond the physiological process of maturation) would further increase the levels predicted (for both FOOD and SCIT exposure) depending on the extent of GFR reduction.

Uncertainty regarding the toxicological assessment arises from the inconsistency of tissue data reported in literature requiring conversion of Al tissue levels reported per dry weight into values referring to the wet weight as predicted by the model. Moreover, we are aware that our assessment based on ULN or critical levels from adults implicitly relies on the assumption that a child is at least not more sensitive to increasing Al concentrations than adults.

In summary, our simulations suggest that Al increase in plasma and tissues from single SCIT treatments is not marginal but appears tolerable considering the worst‐case assumptions applied. Potential risks always need to be weighed against the chance of a curative treatment of an IgE‐mediated allergic disease.

For two scenarios, parallel or lifelong therapy with high Al doses in children and adolescents, it was predicted that a fraction of at least 5% will exceed the normal range of bone Al levels of healthy adults. This increase may reach levels where potential harm to bone metabolism cannot be excluded. Therefore, such treatment decisions should be carefully considered.

Our results imply that, from a toxicological point of view, the limit of 1.25 mg Al per human dose for allergen immunotherapeutics set by the European Pharmacopoiea^8^ is reasonable and could furthermore be seen as a conservative limit for the sum of doses from parallel treatments.

## AUTHOR CONTRIBUTIONS

**Brigitte Keller‐Stanislawski:** Conceptualization; writing – review and editing; supervision. **Wilhelm Huisinga:** Methodology; validation; writing – review and editing. **Gaby Wangorsch:** Conceptualization; methodology; visualization; writing – original draft. **Niklas Hartung:** Methodology; validation; visualization; writing – review and editing. **Karin Weisser:** Conceptualization; writing – original draft.

## FUNDING INFORMATION

This research received no funding.

## CONFLICT OF INTEREST STATEMENT

The authors declare no conflict of interest in relation to this work.

## Supporting information


Figures S1‐S3.
