# Supplementary material for: Model‐informed exploration of the boundaries of safe aluminium exposure from allergen immunotherapy in children
Source: Pediatr Allergy Immunol. 2025 Aug 26;36(8):e70181. doi: 10.1111/pai.70181 (PMC12379567; doi:10.1111/pai.70181)

Supporting information to Weisser et al.

Fig. S1:
Predicted Al levels in bone from birth to adulthood resulting from average dietary exposure in males (A) and females (B) solid line: median; shaded areas: quantiles

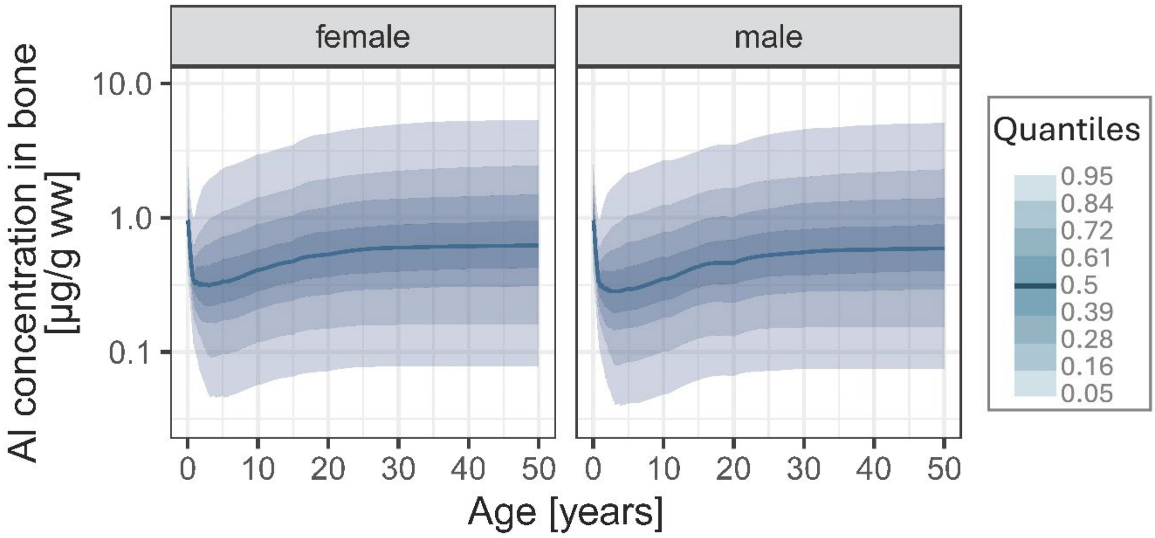

Fig. S2:
Predictions of Al concentration time courses in the kidney following all simulated SCIT scenarios (see Fig. 2 and 3, main text) in addition to the continuous background dietary exposure (solid line: median; shaded areas: quantiles; vertical shaded box: time period of SCIT treatment; horizontal dashed line: upper limit of normal (see Methods); dotted line: median time course of “FOOD ONLY” exposure (upper left panel))

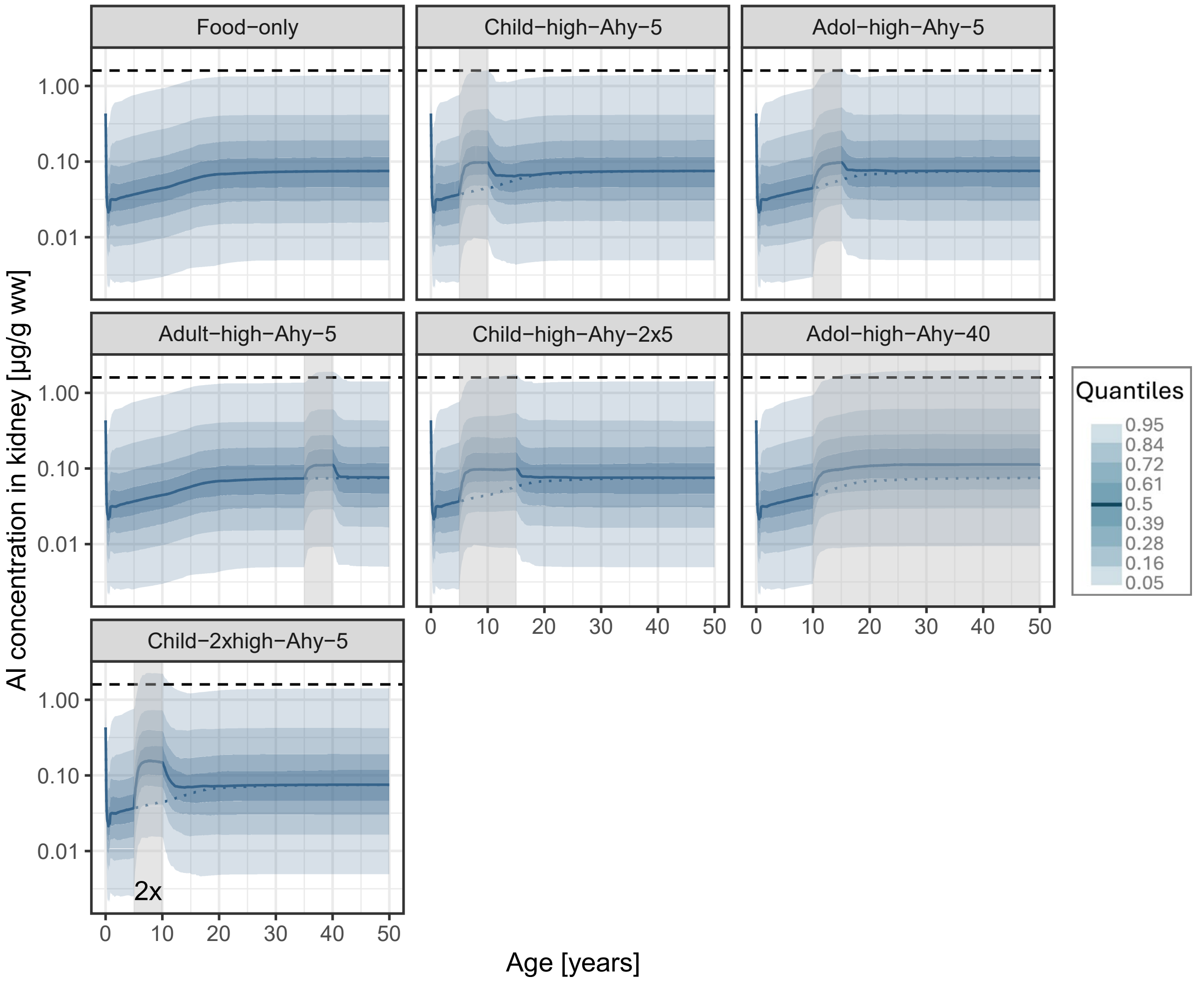

Fig. S3:

Predictions of Al concentration time courses in plasma, bone, and brain following a 5-year SCIT (in situ prepared AH (*Ains)* adjuvant type) during childhood (*child-high-Ains-5*) in addition to the background dietary exposure (solid line: median; shaded areas: quantiles; vertical shaded box: time period of SCIT treatment; horizontal dashed line: upper limit of normal (see Methods); dotted line: median time course of “FOOD ONLY” exposure (see Fig. 1 of the main text))

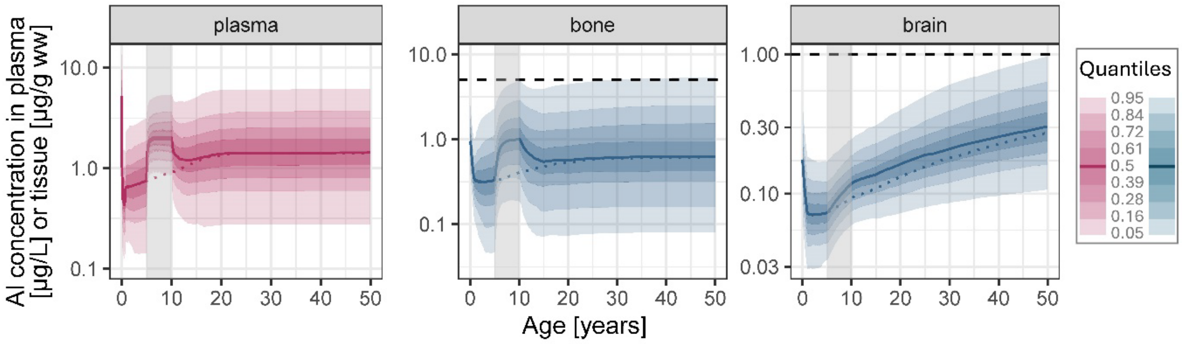

Supplement: Supplementary file 1 — Figures S1‐S3. [file PAI-36-e70181-s001.pdf]
